# Reporting quality of European and Croatian health practice guidelines according to the RIGHT reporting checklist

**DOI:** 10.1186/s13012-018-0828-4

**Published:** 2018-10-29

**Authors:** Ružica Tokalić, Marin Viđak, Ivan Buljan, Ana Marušić

**Affiliations:** Department of Research in Biomedicine and Health, University of Split School of Medicine, Split, Croatia

**Keywords:** Health practice guidelines, Reporting guidelines, RIGHT checklist, Evidence translation

## Abstract

**Background:**

Health practice guidelines (HPGs) are important tools for the translation of evidence into practice. Reporting Items for Practice Guidelines in HealThcare (RIGHT) checklist provides guidance on reporting health practice guidelines (HPGs). We assessed the reporting completeness and quality of a set of national (Croatian) and relevant transnational (European) HPGs.

**Methods:**

The national sample included all HPGs published in the official journal of the Croatian Medical Association in 2014–2016. We searched PubMed to identify relevant European guidelines (*n* = 24). Two independent reviewers assessed the adherence with the items on the RIGHT checklist. Kappa score was used to measure the level of agreement. Frequentist and Bayes statistics Bayes factor (BF_10_) was used to evaluate the differences between the national and transnational HPGs.

**Results:**

Overall, Croatian and European HPGs adhered to less than 50% of RIGHT checklist items. Croatian HPGs reported a median of 14.0 (95% CI 13.0–15.0) RIGHT reporting items, and European counterparts reported a median of 16.0 (95% CI 14.0–17.2) out of the total of 35 checklist items (Mann Whitney *U* test, *P* = 0.048; BF_10_ = 1.543). European HPGs were better than Croatian HPGs in reporting stakeholder involvement and values and preferences (BF_10_ = 80.63), as well as describing the implications of costs and resources (BF_10_ = 55.15). Croatian HPGs better reported HPGs specified aims (BF_10_ = 16.90), primary intended users (BF_10_ = 8.70), and sources of funding (BF_10_ = 122.90). Most insufficiently reported items for both HPG sets were defining the guideline questions and clear outcomes, quality assurance, management of funding and conflicts of interest, and guideline limitations.

**Conclusions:**

Important methodological details are missing from most published HPGs at national and transnational levels. To ensure better quality and adequate use of HPGs, reporting guidelines should be endorsed and used by developers and users alike.

**Electronic supplementary material:**

The online version of this article (10.1186/s13012-018-0828-4) contains supplementary material, which is available to authorized users.

## Background

Health practice guidelines (HPGs) are “statements that include recommendations intended to optimize patient care. They are informed by a systematic review of evidence and an assessment of the benefits and harms of alternative care options” [1]. Guidelines that can be trusted are not only based on a systematic review of best available evidence, but they should be developed by a multidisciplinary panel in a transparent, stakeholder inclusive, documented, and accessible process [1].

HPGs have become a standard in clinical practice and their number has been on a continual increase. PubMed search for MeSH terms “Practice Guideline [Publication Type]” or “Practice Guidelines as Topic” returned 126,573 publications on May 2, 2018, with 23,976 publications related to Europe. Despite this high production rate of and interest in HPGs, there is a great variation in their development, quality, implementation, and use, as demonstrated by the recent overview of the framework for HPGs development at the national level in Europe [2]. In many countries, the development of HPGs is tasked to different specialty/professional societies [2], which thus have a central role in translation of evidence to local practice, most often in the local language. The local guidelines are usually published in national journals or on the websites of professional societies, which serve as dissemination outlets for the HPGs.

Quality of reporting has been recognized as a key measure for successful translation of evidence to practice, reducing overall waste in research and helping in eliminating non-replicable studies [3]. Whereas a number of guidelines for reporting research results have been developed for different types of research [4], the quality of reporting of HPGs is not well known, primarily because there have been no tools specially developed for HPGs. Experience from other fields shows that reporting guidelines, such as Consolidated Standards of Reporting Trials (CONSORT) for randomized controlled trials and STARD for diagnostic studies, are effective in improving completeness of reporting in their respective fields [5–7]. Recently, two reporting checklists have become available for HPGs: Appraisal of Guidelines, Research and Evaluation (AGREE) Reporting Checklist [8] and Reporting Items for Practice Guidelines in HealThcare (RIGHT) [9]. Whereas the AGREE checklist was developed on the concept of the AGREE tools for the methodological rigor and transparency of HPGs [10], the RIGHT checklist follows standard reporting guideline approach established by other reporting statements, such as CONSORT and Preferred Reporting Items for Systematic Reviews and Meta-Analyses (PRISMA) [11, 12]. The RIGHT checklist was developed to assist guideline developers in reporting, journal editors and peer reviewers in decision making, and health care practitioners in understanding and implementing guidelines [9]. So far, RIGHT checklist has only been used to evaluate Chinese guidelines for sepsis and for acupuncture [13, 14].

The aim of our study was to assess and compare the quality of reporting in nationally and transnationally produced (European) HPGs by using the RIGHT reporting checklist. We assessed HPGs developed by professional societies in Croatia, a non-English speaking country, and compared them to similar European HPGs produced by transnational professional societies. We included only European HPGs as comparators on the basis of similarity of populations they address, health problems that are explored, and also because Croatian guidelines often rely on, adapt, and cite their European counterparts.

## Methods

### Study design

We used a cross-sectional design to assess a cohort of published Croatian and relevant European guidelines.

### Cohort of Croatian HPGs

In Croatia, most of the guidelines are developed independently by specialty societies gathered around the Croatian Medical Association, except a few nationally developed guidelines, such as ISKRA HPGs concerning the use of antibiotics [15]. HPGs are most often published in *Liječnički vjesnik*, the official journal of the Croatian Medical Association [16]. For this study, we included all HPGs published in *Liječnički vjesnik* from 2014 until March 2017: 26 guidelines (last guideline published in 2016).

### Search strategy for relevant European HPGs

To identify matching European guidelines, we reviewed the bibliography of included documents and searched PubMed and Google. We used the guideline topic as a search term combined with “Europe” and “guideline or recommendation.” Guideline topic included the clinical condition and intervention mentioned in the Croatian HPGs (e.g., type 2 diabetes pharmacotherapy, primary breast cancer diagnosis). Two authors (RT and MV) independently identified guidelines for inclusion, and the final decision was made by consensus.

Some of the Croatian HPGs covered more than one topic, i.e., clinical condition and/or intervention. In those cases, we searched for all topics covered by the guideline and included all relevant European guidelines.

The search for European guidelines was performed in July 2017. We excluded guidelines that were adapted for non-European populations, global or international guidelines, and those unavailable in English or Croatian.

### Data extraction

We used the RIGHT checklist to evaluate the published HPGs. The RIGHT checklist [17] contains 22 requirements organized into 7 sections with a total of 35 items: basic information (6 items), background (8 items), evidence (5 items), recommendations (7 items), review and quality assurance (2 items), funding and declaration and management of interest (4 items), and other information (3 items). Two authors (RT and MV) independently assessed the adherence of HPGs with the RIGHT checklist. In case of conflicting opinions, the third author (AM) was consulted and agreement was reached by consensus. Kappa statistic was used to measure the level of agreement.

### Data analysis

Data are presented as the number of RIGHT checklist items reported in each guideline, as well as number of guidelines that reported individual RIGHT checklist items and their proportion, with 95% confidence interval. Due to the non-normality of distributions, we performed Mann-Whitney *U* test to compare the two groups of HPGs scores overall. For each RIGHT item separately, we calculated the Bayes factor in the contingency table and frequentist chi-square test to evaluate the differences between the two groups. Bayes factor (BF_10_) was calculated assuming a default prior distribution. *P* value < 0.05 and BF_10_ ≥ 3 were considered significant [18]. In cases of conflicting frequentist and Bayesian results of significance, we considered the results of Bayes statistics for final interpretation. Calculations were done using MedCalc Statistical Software version 16.4.3 (MedCalc Software bvba, Ostend, Belgium; https://www.medcalc.org; 2016), and JASP software, version 0.8.6 (https://jasp-stats.org/).

## Results

We reviewed all published Croatian HPGs (*n* = 26) and 24 matching European HPGs. Eleven Croatian HPGs did not have European counterparts, and 3 of them had multiple European counterparts. Because of these discrepancies, we did not do a matched analysis. Kappa coefficient for individual 35 items of RIGHT checklist ranged from 0.76 to 1.0. All of the European guidelines were produced by transnational specialty societies, and most of Croatian guidelines were produced by national specialty societies (23 out of 26; 3 were produced by individual authors, independently from specialty societies; 1 of those declared to be supported by professional societies). All of the guidelines were produced or updated in 2014 or later. The full list of guidelines and individual results of the adherence to the RIGHT checklist are available in the Additional file 1.

Overall, Croatian and European HPGs adhered to less than 50% of RIGHT checklist items. Croatian HPGs reported a median of 14.0 (95% CI 13.0–15.0) RIGHT reporting items. Their European counterparts reported a median of 16.0 (95% CI 14.0–17.2) out of the total of 35 checklist items. The difference was not significant (*P* = 0.048, Mann-Whitney *U* test, BF_10_ = 1.543). The lowest total number of addressed RIGHT items was 11 (31%) (Croatian HPGs on melanoma and cystine urolithiasis), and the highest was 22 (63%, European resuscitation guideline).

The results of adherence to individual RIGHT checklist items are presented in Tables 1, 2, and 3. For nine checklist items, both groups of HPGs had a satisfactory level of reporting (≥ 80% of HPGs in each group adhered to the checklist item): item 1a—clear guideline titles, described as mentioning the focus of the guideline; item 1c—defining it as guidelines or recommendations in the title; item 4—providing an address of a corresponding author; item 9a—lists of authors and their affiliations; item 5*—*description of basic epidemiology of health problems; item 7a—description of primary populations addressed by the HPG; item 13a—clear and actionable recommendations; item 13b—separate recommendations for important subgroups; and item 13c—indicated strengths of recommendations and certainty of evidence.

**Table 1:** Adherence of health practice guidelines (HPGs) to RIGHT checklist items addressing the domains of basic information and background (total number and percentage with 95% confidence intervals (CI) of addressed items)

RIGHT checklist item	Croatian HPGs (*n* = 26)	European HPGs (*n* = 24)	P^a^	BF_10_^b^
Domain 1: Basic information				
Title/subtitle	Total number (%, 95% CI)			
1a—Identify the report as a guideline, that is, with “guideline(s)” or “recommendation(s)” in the title.	26 (100.0, 87.3–100.0)	22 (91.7, 74.2–97.6)	0.130	0.34
1b—Describe the year of publication of the guideline.	1 (3.8, 2.0–21.6)	3 (12.5, 4.0–31.0)	0.260	0.33
1c—Describe the focus of the guideline, such as screening, diagnosis, treatment, management, prevention or others.	26 (100.0, 87.3–100.0)	21 (87.5, 69.0–95.7)	0.063	0.73
Executive summary				
2—Provide a summary of the recommendations contained in the guideline.	10 (38.4, 20.9–59.3)	17 (70.8, 50.8–85.1)	0.022	4.44
Abbreviations and acronyms				
3—Define new or key terms, and provide a list of abbreviations and acronyms if applicable.	4 (15.3, 5.0–35.7)	4 (16.7, 6.7–35.9)	0.902	0.26
Corresponding developer				
4—Identify at least one corresponding developer or author who can be contacted about the guideline.	26 (100.0, 87.3–100.0)	23 (95.8, 79.8–99.3)	0.293	0.16
Domain 2: Background				
Brief description of the health problem(s)				
5—Describe the basic epidemiology of the problem, such as the prevalence/incidence, morbidity, mortality, and burden (including financial) resulting from the problem.	23 (88.4, 68.7–96.9)	22 (91.7, 74.2–97.6)	0.706	0.22
Aim(s) of the guideline and specific objectives				
6—Describe the aim(s) of the guideline and specific objectives, such as improvements in health indicators (e.g., mortality and disease prevalence), quality of life, or cost savings.	20 (76.9, 55.9–90.2)	9 (37.5, 21.2–57.3)	0.005	16.90
Target population(s)				
7a—Describe the primary population(s) that is addressed by the recommendation(s) in the guideline.	22 (84.6, 64.3–94.9)	22 (91.7, 74.2–97.6)	0.443	0.29
7b—Describe any subgroups that are given special consideration in the guideline.	20 (76.9, 55.9–90.2)	15 (62.5, 42.7–78.8)	0.266	0.57
End-users and settings				
8a—Describe the intended primary users of the guideline (such as primary care providers, clinical specialists, public health practitioners, program managers, and policy-makers) and other potential users of the guideline.	12 (46.2, 27.1–66.3)	3 (12.5, 4.0–31.0)	0.009	8.70
8b—Describe the setting(s) for which the guideline is intended, such as primary care, low- and middle-income countries, or in-patient facilities.	8 (30.7, 15.1–51.9)	4 (16.7, 6.7–35.9)	0.243	0.55
Guideline development groups				
9a—Describe how all contributors to the guideline development were selected and their roles and responsibilities (e.g., steering group, guideline panel, external reviewer, systematic review team, and methodologists).	4 (15.3, 0.5 to 35.7)	3 (12.5, 4.0–31.0)	0.769	0.25
9b—List all individuals involved in developing the guideline, including their title, role(s) and institutional affiliation(s).	26 (100.0, 87.3–100.0)	23 (95.8, 79.8–99.3)	0.293	0.16

^a^Frequentist chi-square test

^b^Bayesian 2 × 2 contingency table

**Table 2:** Adherence of health practice guidelines (HPGs) to RIGHT checklist items addressing the domains of evidence and recommendations (total number and percentage with 95% confidence intervals (CI) of addressed items)

RIGHT checklist item	Croatian HPGs (*n* = 26)	European HPGs (*n* = 24)	P^a^	BF_10_^b^
Domain 3: Evidence				
Healthcare questions	Total number (%, 95% CI)			
10a—State the key questions that were the basis for the recommendations in PICO (population, intervention, comparator, and outcome) or other format as appropriate.	1 (3.8, 0.2–21.6)	0 (0.0, 0.0–13.8)	0.332	0.15
10b—Indicate how the outcomes were selected and sorted.	1 (3.8, 0.2–21.6)	0 (0.0, 0.0–13.8)	0.332	0.14
Systematic reviews				
11a—Indicate whether the guideline is based on new systematic reviews done specifically for this guideline or whether existing systematic reviews were used.	3 (11.5, 3.0–31.3)	3 (12.5, 4.0–31.0)	0.917	0.23
11b—If the guideline developers used existing systematic reviews, reference these and describe how those reviews were identified and assessed (provide the search strategies and the selection criteria, and describe how the risk of bias was evaluated) and whether they were updated.	0 (0.0, 0.0–16.2)	3 (12.5, 4.0–31.0)	0.063	0.73
Assessment of the certainty of the body of evidence				
12—Describe the approach used to assess the certainty of the body of evidence.	19 (73.1, 51.9–87.7)	18 (75.0, 55.1–88.0)	0.877	0.30
Domain 4: Recommendations				
Recommendations				
13a—Provide clear, precise, and actionable recommendations.	26 (100.0, 87.3–100.0)	24 (100.0, 86.2–100.0)	0.777	NA
13b—Present separate recommendations for important subgroups if the evidence suggests that there are important differences in factors influencing recommendations, particularly the balance of benefits and harms across subgroups.	25 (96.2, 78.4–99.8)	22 (91.7, 74.2–97.6)	0.504	0.21
13c—Indicate the strength of recommendations and the certainty of the supporting evidence.	21 (80.7, 60.2–92.7)	21 (87.5, 69.0–95.7)	0.517	0.31
Rationale/explanation for recommendations				
14a—Describe whether values and preferences of the target population(s) were considered in the formulation of each recommendation. If yes, describe the approaches and methods used to elicit or identify these values and preferences. If values and preferences were not considered, provide an explanation.	2 (7.7, 1.3–26.6)	12 (50.0, 31.4–68.6)	< 0.001	80.63
14b—Describe whether cost and resource implications were considered in the formulation of recommendations. If yes, describe the specific approaches and methods used (such as cost effectiveness analysis) and summarize the results. If resource issues were not considered, provide an explanation.	0 (0.0, 0.0–16.2)	8 (33.3, 17.9–53.3)	< 0.001	55.15
14c—Describe other factors taken into consideration when formulating the recommendations, such as equity, feasibility and acceptability.	2 (7.7, 1.3–26.6)	6 (25.0, 12.0–44.9)	0.095	0.93
Evidence to decision processes				
15—Describe the processes and approaches used by the guideline development group to make decisions, particularly the formulation of recommendations (such as how consensus was defined and achieved and whether voting was used).	9 (34.6, 17.9–55.6)	4 (16.7, 6.7–35.9)	0.148	0.81

*NA* not applicable

^a^Frequentist chi square test

^b^Bayesian 2 × 2 contingency table

**Table 3:** Adherence of health practice guidelines (HPGs) to RIGHT checklist items addressing the domains of review and quality assurance, funding, declaration and management of interest, and other information (total number and percentage with 95% confidence intervals (CI) of addressed items)

RIGHT checklist item	Croatian HPGs (*n* = 26)	European HPGs (*n* = 24)	P^a^	BF_10_^b^
Domain 5: Review and quality assurance				
External review	Total number (%, 95% CI)			
16—Indicate whether the draft guideline underwent independent review and, if so, how this was executed and the comments considered and addressed.	1 (3.8, 0.2–21.6)	10 (41.7, 24.5–61.2)	< 0.001	55.35
Quality assurance				
17—Indicate whether the guideline was subjected to a quality assurance process. If yes, describe the process.	1 (3.8, 0.2–21.6)	3 (12.5, 4.0–31.0)	0.260	0.33
Domain 6: Funding, declaration and management of interest				
Funding source(s) and role(s) of the funder				
18a—Describe the specific sources of funding for all stages of guideline development.	18 (69.2, 48.1–84.9)	5 (20.8, 9.2–40.5)	< 0.001	122.90
18b—Describe the role of funder(s) in the different stages of guideline development and in the dissemination and implementation of the recommendations.	2 (7.7, 1.3–26.6)	2 (8.3, 2.3–25.9)	0.933	0.19
Declaration and management of interest				
19a—Describe what types of conflicts (financial and non-financial) were relevant to guideline development.	2 (7.7, 1.3–26.6)	22 (91.7, 74.2–97.6)	< 0.001	1.02 × 10^8^
19b—Describe how conflicts of interest were evaluated and managed and how users of the guideline can access the declarations.	1 (3.8, 0.2–21.6)	1 (4.2, 0.7–20.2)	0.954	0.15
Domain 7: Other information				
Access				
20—Describe where the guideline, its appendices, and other related documents can be accessed.	1 (3.8, 0.2–21.6)	3 (12.5, 4.0–31.0)	0.260	0.33
Suggestions for further research				
21—Describe the gaps in the evidence and/or provide suggestions for future research.	12 (46.2, 27.1–66.3)	23 (95.8, 79.8–99.3)	< 0.001	733.7
Limitations of the guideline				
22—Describe any limitations in the guideline development process (such as the development groups were not multidisciplinary or patients’ values and preferences were not sought), and indicate how these limitations might have affected the validity of the recommendations.	1 (3.8, 0.2–21.6)	1 (4.2, 0.7–20.2)	0.954	0.15

^a^Frequentist chi square test

^b^Bayesian 2 × 2 contingency table

For 12 out of 35 RIGHT items, both sets of HPGs had poor reporting (≤ 20% of HPGs in each group adhered to the checklist item): item 9a—description of roles and responsibilities of authors and contributors; item 10a—a clear and structured healthcare question in PICO or other appropriate format; item 10b—clearly defined outcomes; items 11a and 11b—process of selection of systematic reviews; item 17—description of quality assurance process; item 18b—reporting on the roles of funding bodies in the development; item 19b—management of conflicts of interest; item 22—description of limitations of the guidelines, such as risk of bias, conflicts of interest or lack of patient involvement; and item 20—availability of guidelines and additional documents.

European guidelines were significantly better in providing a HPG summary (item 2; BF_10_ = 4.44), reporting stakeholder involvement and values and preferences (item 14a; BF_10_ = 80.63), as well as describing the implications of costs and resources (item 14b; BF_10_ = 55.15, Table 2). They also reported better than Croatian HPGs on existing conflicts of interest (item 16; BF_10_ = 55.35), external reviews (item 19a; BF_10_ = 1.02 × 10^8^), and on sufficiency of evidence for recommendations or provided suggestions for future research (item 21; BF_10_ = 733.70).

Croatian HPGs were significantly better in reporting specified aims of the guideline (item 6, BF_10_ = 16.90), their primary intended users (item 8a, BF_10_ = 8.70), and sources of funding (item 18a, BF_10_ = 122.90).

For item 12 (description of the approach used to assess the certainty of the body of evidence), the Croatian and European HPGs sets had similar adherence (73.1%, 95% CI 51.9–87.7 for Croatian, and 75.0%, 95% CI 55.1–88.0 for European HPGs; BF_10_ = 0.30). We also extracted the information on what sort of approach was used in the HPGs. Grading of Recommendations, Assessment, Development and Evaluation (GRADE) handbook [19] and Oxford Centre for Evidence Based Medicine levels of evidence [20] were predominant approaches in Croatian HPGs (53.8% of HPGs), but were less frequent in the European HPGs set (16.7% of HPGs). European HPGs mostly mentioned adapted Infectious Diseases Society of America–United States Public Health Service Grading System [21] (45.8% of HPGs). Other grading systems, such as American College of Chest Physicians (ACCP) modified GRADE system [22] or Scottish Intercollegiate Guidelines Network (SIGN) guide [23], were mentioned in 7.7% of Croatian and 12.50% of European HPGs. Some HPGs were unclear about the grading systems, mentioning the levels of evidence but not referencing any grading systems (23.1% of Croatian and 16.7% of European HPGs). Finally, 15.4% of Croatian and 8.3% of European HPGs did not mention or imply the use of any grading systems for levels of evidence.

## Discussion

Our study showed that both national and transnational HPGs were poorly reported in journals, with adequate presentation for fewer than 50% of necessary reporting items. This is, to the best of our knowledge, the first evaluation of HPGs in Europe using the RIGHT reporting checklist, developed for guidance in reporting guidelines in healthcare, including clinical and public health areas. It is important to keep in mind that the RIGHT checklist is not a guide for development nor a tool for assessment of methodological quality of HPGs, but a tool for checking the clarity of presentation of the HPGs to a wide audience, including both developers and readers, so that HPGs could be adequately understood and implemented in practice [9].

Our finding of inadequate reporting of HPGs matches previous observations of poor methodological quality of HPGs produced by specialty societies [24, 25]. There was no difference between the quality of reporting of national HPGs, which are directly implemented in practice in individual health care systems, and those produced by transnational societies, which put emphasis on multidisciplinary collaboration and have standard operating procedures for guideline development [26].

The relatively small sample size is the most important limitation of our study. The sample size was determined by the cohort of available Croatian HPGs. We did not include HPGs published earlier than 2014 as they were not numerous and their presentation was of poor quality (our unpublished observation). The small sample size was also the reason why we based the interpretation of results on the results of Bayes and not frequentist statistics. The advantages of coherency, independency of the intention with which data are collected, and minimum bias toward null hypothesis made Bayesian inference valid for small sample sizes [27]. It is also important to keep in mind that we analyzed national HPGs in a single country, so the results may not be generalizable to the whole population of nationally produced guidelines. However, the similarity of findings for Croatian and matching transnational guidelines indicates that the level of reporting quality is similar across professional societies in Europe. Finally, it is important to keep in mind that the RIGHT checklist does not evaluate the validity of the actual recommendations in the HPGs, but only the quality of their publicly available presentation.

The analysis of compliance with individual reporting items demonstrated that there was an overall insufficient emphasis on providing clear aims and a summary of recommendations. Guideline developers failed to provide clear instructions on how the guidelines are intended to be used and by whom. In this way, basic principles of evidence-based medicine had not been addressed because there was little information on the users of HPGs, the purpose of HPGs, and acknowledging barriers and constraints that may limit their implementation [28].

Both national and transnational sets of HPGs adequately reported on strengths of recommendations and certainty of supporting evidence, but details on how the evidence was searched for and evaluated were unsatisfactory. Established grading systems, such as GRADE and Oxford EBM, were used in half or less than a half of the analyzed HPGs, and 22% of HPGs were unclear about grading evidence. This means that current HPGs do not satisfy the preference of clinicians for recommendations that have clearly presented processes behind them and provide evidence summaries [29, 30]. Clear and transparent reporting should also help address problems such as relying on lower levels of evidence than stated in HPGs [31], relying on low levels of evidence to form recommendations [32], and inadequate presentation of evidence grading [33].

In national HPGs, reporting on the inclusion of patient values and preferences in the making of recommendations was poor, and reporting cost and resource considerations was almost non-existent. This reflects generally unsatisfactory involvement of the patients in guideline development [34], as well as integration of evidence on patient preferences [35], even though the involvement of patients and other stakeholders is especially important for development and adaptation of HPGs oriented at lower- and middle-income populations, which often have limited resources and different patient experiences and priorities [36, 37].

We also found that reporting of conflicts of interest (COI) was worse in Croatian than in European HPGs, but that both groups of guidelines showed low adherence to providing declarations on the conflicts of interest and elaborating policies regarding their management. Available data show that the prevalence of conflicts of interest in guideline development is high [38], and that conflicts of interest can influence interpretation and recommendations of treatments [39], as well as definitions of diseases [40]. Croatia is a country that follows the practice of the European Federation of Pharmaceutical Industries and Associations (EFPIA) Code for disclosure of financial transfers [41], but does not have a national legislation that requires disclosure on a central platform, such as Denmark [42]. Even in systems with mandatory conflict of interest declaration, like Denmark, there is a discrepancy between disclosures in the national register and self-reporting in publications [42]. In Croatia, only 11% of physicians made their individual data available in 2016, when the first report from the Innovative Pharmaceutical Initiative, an association that gathers 33 pharmaceutical companies that cover 60% of Croatian pharmaceutical market, was published [43].

## Conclusion

Our study provides evidence to recommend the use of reporting checklists to guideline developers both at national and transnational levels. A variety of tools have been developed to help translation of evidence to reliable and high-quality recommendations, including GRADE system for rating quality of evidence [44], AGREE tools for guideline methodology [10], checklists for development, implementation, evaluation, and quality [45, 46]. Without complete and transparent reporting, there is no way of knowing how good and how appropriate HPGs are for use in specific clinical circumstances. In times of overwhelming production of guidelines, which are supposed to help the end-user in finding and summarizing the best evidence [47], it is particularly important to achieve clarity and enable informed decisions. In future research, the usefulness of RIGHT reporting guideline should be tested in actual health practice settings and they should be compared with similar reporting guidelines, such as AGREE Reporting Checklist [8]. Endorsement of reporting guidelines for HPGs, such as RIGHT, by professional associations, health websites, and journals would contribute to the effort of transparency for developers and to the aim of adequate implementation for users of guidelines. Only then can we hope to achieve the goal of full transparency, comprehension, and availability of information that concerns a variety of stakeholders.

## Additional file

Additional file 1: File contains **Table S1. and S2**, which contain full lists of assessed HPGs and number of RIGHT checklist items reported in each individual HPG. (DOCX 23 kb)

## Abbreviations

AGREE: Appraisal of Guidelines for Research and Evaluation
CONSORT: Consolidated Standards of Reporting Trials
GRADE: Grading of Recommendations Assessment, Development and Evaluation
HPG: Health practice guideline
PRISMA: Preferred Reporting Items for Systematic Reviews and Meta-Analyses
RIGHT: Reporting Items for Practice Guidelines in HealThcare
SIGN: Scottish Intercollegiate Guidelines Network
STARD: Standards for Reporting of Diagnostic Accuracy Studies
