# Supplementary material for: Reporting quality of European and Croatian health practice guidelines according to the RIGHT reporting checklist
Source: Implement Sci. 2018 Oct 29;13:135. doi: 10.1186/s13012-018-0828-4 (PMC6206632; doi:10.1186/s13012-018-0828-4)
Supplement: Supplementary file 1 — File contains Table S1. and S2, which contain full lists of assessed HPGs and number of RIGHT checklist items reported in each individual HPG. (DOCX 23 kb) [file 13012_2018_828_MOESM1_ESM.docx]

**Table S1.** Number of RIGHT checklist items reported in individual Croatian health practice guidelines

| Year of publication | Guideline | Number (%) of RIGHT checklist elements present (max. 35) |
| --- | --- | --- |
| 2016 | Hunyadi-Antičević S, Protić A, Patrk J, Filipović-Grčić B, Puljević D, Majhen-Ujević R, et al. Smjernice za reanimaciju Europskog vijeća za reanimatologiju 2015. godine. Liječnički Vjesnik 2016;138:305–321. | 15 (42.9%) |
| 2016 | Rahelić D, Altabas V, Bakula M, Balić S, Balint I, Bergman Marković B, et al. Hrvatske smjernice za farmakološko liječenje šećerne bolesti tipa 2. Liječnički Vjesnik 2016;138:1–21. | 12 (34.3%) |
| 2016 | Herceg D, Štulhofer Buzina D, Čeović R, Dotlić S, Ilić I, Smuđ Orehovec S, et al. Kliničke preporuke hrvatskog društva za internističku onkologiju HLZ-a za dijagnozu, liječenje i praćenje bolesnika/ca oboljelih od melanoma kože. Liječnički Vjesnik 2016;138:22–29. | 11 (31.4%) |
| 2016 | Bišof V, Juretić A, Stančić-Rokotov D, Rustemović N, Miletić D, Boban M, et al. Kliničke preporuke za dijagnozu, liječenje i praćenje bolesnika oboljelih od raka jednjaka i ezofagogastričnog prijelaza. Liječnički Vjesnik 2016;138:233–239. | 14 (40.0%) |
| 2016 | Šeparović R, Silovski T, Dedić Plavetić N, Šerman A, Grubišić Čabo F, Kardum Fucak I, et al. Praćenje onkoloških bolesnika – kliničke preporuke hrvatskog društva za internističku onkologiju HLZ-a 1. dio: rak dojke, rak tijela maternice, rak vrata maternice, rak jajnika. Liječnički Vjesnik 2016;138:63–68. | 17 (48.6%) |
| 2016 | Gnjidić M, Vojnović Ž, Čonkaš M, Grubišić Čabo F, Belev B, Budisavljević A, et al. Praćenje onkoloških bolesnika – kliničke preporuke hrvatskog društva za internističku onkologiju HLZ-a II. dio: rak bubrega, rak mokraćnog mjehura, rak prostate, rak testisa. Liječnički Vjesnik 2016;138:167–172. | 17 (48.6%) |
| 2016 | Dedić Plavetić N, Kelemenić Dražin R, Dobrila Dintinjana R, Radić M, Prejac J, Mišetić Dolić Z, et al. Praćenje onkoloških bolesnika – kliničke preporuke hrvatskog društva za internističku onkologiju HLZ-a III. dio: neuroendokrine neoplazme, hepatocelularni karcinom, rak gušterače, rak žučnih vodova. Liječnički Vjesnik 2016;138:173–178. | 16 (45.7%) |
| 2016 | Bašić-Jukić N, Pavlović D, Šmalcelj R, Tomić-Brzac H, Orlić L, Radić J, et al. Smjernice za prevenciju, praćenje i liječenje poremećaja koštano-mineralnog metabolizma u bolesnika s kroničnom bubrežnom bolesti. Liječnički Vjesnik 2016;138:107–120. | 18 (51.4%) |
| 2016 | Vranešić Bender D, Giljević Z, Kušec V, Laktašić Žerjavić N, Bošnjak Pašić M, Vrdoljak E. Smjernice za prevenciju, prepoznavanje i liječenje nedostatka vitamina D u odraslih. Liječnički Vjesnik 2016;138:121–132. | 15 (42.9%) |
| 2016 | Arbanas G, Jurin T, Mozetič V, Mimica Matanović S, Rožman J, Markić D, et al. Prve hrvatske smjernice za dijagnostiku, liječenje i praćenje osoba s prijevremenom ejakulacijom. Liječnički Vjesnik 2016;138:321–327. | 19 (54.05) |
| 2015 | Maslovara S, Butković-Soldo S, Drviš P, Roje-Bedeković M, Trotić R, Branica S, et al. Hrvatske smjernice za dijagnostiku i liječenje benignoga paroksizmalnog pozicijskog vertiga (BPPV-a). Liječnički Vjesnik 2015;137:335–342. | 15 (42.9%) |
| 2015 | Bišof V, Juretić A, Trivanović D, Dobrila Dintinjana R, Šarčević B, Jakić-Razumović J, et al. Kliničke preporuke za dijagnozu, liječenje i praćenje bolesnika oboljelih od raka nepoznata primarnog podrijetla. Liječnički Vjesnik 2015;137:65–69. | 12 (34.3%) |
| 2015 | Šeparović R, Ban M, Silovska T, Beketić Orešković L, Soldić Ž, Podolski P, et al. Kliničke upute hrvatskoga onkološkog društva za dijagnozu, liječenje i praćenje bolesnica/ka oboljelih od invazivnog raka dojke. Liječnički Vjesnik 2015;137:143–149. | 14 (40.0%) |
| 2015 | Mišir Krpan A, Juretić A, Boban M, Omrčen T, Paladino J, Hajnšek S, et al. Kliničke upute za dijagnozu, liječenje i praćenje odraslih bolesnika oboljelih od glioma središnjega živčanog sustava. Liječnički Vjesnik 2015;137:343–347. | 14 (40.0%) |
| 2015 | Pećin I, Muačević-Katanec D, Šimić I, Fumić K, Potočki K, Šućur N, et al. Pompeova bolest - smjernice za dijagnozu i liječenje odraslih bolesnika. Liječnički Vjesnik 2015;137:216–218. | 13 (37.1%) |
| 2015 | Bašić-Jukić N, Radić J, Klarić D, Jakić M, Vujičić B, Gulin M, et al. Preporuke za praćenje, prevenciju i liječenje proteinsko-energijske pothranjenosti u bolesnika s kroničnom bubrežnom bolesti. Liječnički Vjesnik 2015;137:1–8. | 14 (40.0%) |
| 2015 | Muačević-Katanec D, Pećin I, Šimić I, Fumić K, Potočki K, Šućur N, et al. Smjernice za liječenje mukopolisaharidoze (MPS) VI u odraslih bolesnika. Liječnički Vjesnik 2015;137:213–215. | 12 (34.0%) |
| 2014 | Kuveždić H, Šimunović D, Mrazovac D, Librenjak D, Oguić R, Jelaković B, et al. Cistinska urolitijaza: preporuke za dijagnostiku, liječenje i prevenciju recidiva. Liječnički Vjesnik 2014;136:68–72. | 11 (31.4%) |
| 2014 | Merkler M, Pećin I, Šimić I, Muačević-Katanec D, Šućur N, Reiner Ž, et al. Fabryjeva bolest smjernice za dijagnozu i liječenje odraslih bolesnika. Liječnički Vjesnik 2014;136:133–135. | 13 (37.1%) |
| 2014 | Merkler M, Šimić I, Pećin I, Muačević-Katanec D, Šućur N, Reiner Ž, et al. Gaucherova bolest smjernice za dijagnozu i liječenje odraslih bolesnika. Liječnički Vjesnik 2014;136:130–133. | 13 (37.1%) |
| 2014 | Zelić M, Vranešić Bender D, Ljubas Kelečić D, Župan Ž, Cicvarić T, Maldini B, et al. Hrvatske smjernice za perioperativnu enteralnu prehranu kirurških bolesnika. Liječnički Vjesnik 2014;136:179–185. | 13 (37.1%) |
| 2014 | Katičić M, Duvnjak M, Filipec Kanižaj T, Krznarić Ž, Marušić M, Mihaljević S, et al. Hrvatski postupnik za dijagnostiku i terapiju infekcije helicobacterom pylori. Liječnički Vjesnik 2014;136:1–17. | 14 (40.0%) |
| 2014 | Katičić M, Banić M, Crnčević Urek M, Gašparov S, Krznarić Ž, Prskalo M, et al. Hrvatski postupnik za prevenciju želučanog raka eradikacijom infekcije Helicobacterom pylori. Liječnički Vjesnik 2014;136:59–68. | 13 (37.1%) |
| 2014 | Houra K, Ledić D, Kvesić D, Perović D, Radoš I, Kapural L. Prve hrvatske smjernice za dijagnostiku i liječenje bolnih stanja vratne i prsne kralježnice minimalno invazivnim postupcima. Liječnički Vjesnik 2014;136:245–252. | 20 (57.1%) |
| 2014 | Stipić Marković A, Rožmanić V, Anić B, Aberle N, Račić G, Novak S, et al. Smjernice za dijagnostiku i liječenje hereditarnog angioedema. Liječnički Vjesnik 2014;136:117–129. | 15 (42.9%) |
| 2014 | Gornik I, Rahelić D, Husedžinović I, Gašparović V, Ivanović D, Krznarić Ž, et al. Smjernice za zbrinjavanje hiperglikemije u odraslih hospitaliziranih bolesnika. Liječnički Vjesnik 2014;136:315–323. | 16 (45.7%) |

**Table S2** Number of RIGHT checklist items reported in individual European health practice guidelines

| No. | Guideline | Number (%) of elements present (max. 35) |
| --- | --- | --- |
| 1 | Monsieurs KG, Nolan JP, Bossaert LL, Greif R, Maconochie IK, Nikolaou NI, et al. European Resuscitation Council Guidelines for Resuscitation 2015: Section 1. Executive summary. Resuscitation. 2015;95:1-80. | 22 (62.9%) |
| 2 | Inzucchi SE, Bergenstal RM, Buse JB, Diamant M, Ferrannini E, Nauck M, et al. Management of hyperglycaemia in type 2 diabetes, 2015: a patient-centred approach. Update to a Position Statement of the American Diabetes Association and the European Association for the Study of Diabetes. Diabetologia. 2015;58(3):429-42. | 18 (51.4%) |
| 3 | Garbe C, Peris K, Hauschild A, Saiag P, Middleton M, Spatz A, et al. Diagnosis and treatment of melanoma. European consensus-based interdisciplinary guideline--Update 2012. European journal of cancer. 2012;48(15):2375-90. | 16 (45.7%) |
| 4 | Lordick F, Mariette C, Haustermans K, Obermannova R, Arnold D. Oesophageal cancer: ESMO Clinical Practice Guidelines for diagnosis, treatment and follow-up. Annals of oncology. 2016;27 Suppl. 5:v50-v7. | 16 (45.7%) |
| 5 | Senkus E, Kyriakides S, Ohno S, Penault-Llorca F, Poortmans P, Rutgers E, et al. Primary breast cancer: ESMO Clinical Practice Guidelines for diagnosis, treatment and follow-up. Annals of oncology. 2015;26 Suppl. 5:v8-30. | 15 (42.9%) |
| 6 | Colombo N, Preti E, Landoni F, Carinelli S, Colombo A, Marini C, et al. Endometrial cancer: ESMO Clinical Practice Guidelines for diagnosis, treatment and follow-up. Annals of oncology. 2013;24 Suppl. 6:vi33-8. | 13 (37.1%) |
| 7 | Colombo N, Carinelli S, Colombo A, Marini C, Rollo D, Sessa C. Cervical cancer: ESMO Clinical Practice Guidelines for diagnosis, treatment and follow-up. Annals of oncology. 2012;23 Suppl 7:vii27-32. | 12 (34.3%) |
| 8 | Ledermann JA, Raja FA, Fotopoulou C, Gonzalez-Martin A, Colombo N, Sessa C. Newly diagnosed and relapsed epithelial ovarian carcinoma: ESMO Clinical Practice Guidelines for diagnosis, treatment and follow-up. Annals of oncology. 2013;24 Suppl 6:vi24-32. | 14 (40.0%) |
| 9 | Escudier B, Porta C, Schmidinger M, Rioux-Leclercq N, Bex A, Khoo V, et al. Renal cell carcinoma: ESMO Clinical Practice Guidelines for diagnosis, treatment and follow-up. Annals of oncology. 2016;27 Suppl 5:v58-v68. | 16 (45.7%) |
| 10 | Bellmunt J, Orsola A, Leow JJ, Wiegel T, De Santis M, Horwich A. Bladder cancer: ESMO Practice Guidelines for diagnosis, treatment and follow-up. Annals of oncology. 2014;25 Suppl 3:iii40-8. | 15 (42.9%) |
| 11 | Parker C, Gillessen S, Heidenreich A, Horwich A. Cancer of the prostate: ESMO Clinical Practice Guidelines for diagnosis, treatment and follow-up. Annals of oncology. 2015;26 Suppl 5:v69-77. | 14 (40.0%) |
| 12 | Albers P, Albrecht W, Algaba F, Bokemeyer C, Cohn-Cedermark G, Fizazi K, et al. Guidelines on Testicular Cancer: 2015 Update. European urology. 2015;68(6):1054-68. | 17 (48.6%) |
| 13 | Oberg K, Knigge U, Kwekkeboom D, Perren A. Neuroendocrine gastro-entero-pancreatic tumors: ESMO Clinical Practice Guidelines for diagnosis, treatment and follow-up. Annals of oncology. 2012;23 Suppl 7:vii124-30. | 12 (34.3%) |
| 14 | Verslype C, Rosmorduc O, Rougier P. Hepatocellular carcinoma: ESMO-ESDO Clinical Practice Guidelines for diagnosis, treatment and follow-up. Annals of oncology. 2012;23 Suppl 7:vii41-8. | 14 (40.0%) |
| 15 | Seufferlein T, Bachet JB, Van Cutsem E, Rougier P. Pancreatic adenocarcinoma: ESMO-ESDO Clinical Practice Guidelines for diagnosis, treatment and follow-up. Annals of oncology. 2012;23 Suppl 7:vii33-40. | 14 (40.0%) |
| 16 | Valle JW, Borbath I, Khan SA, Huguet F, Gruenberger T, Arnold D. Biliary cancer: ESMO Clinical Practice Guidelines for diagnosis, treatment and follow-up. Annals of oncology. 2016;27 Suppl 5:v28-v37. | 16 (45.7%) |
| 17 | Hatzimouratidis K, Eardley I, Giuliano F, Moncada I, Salonia A. Guidelines on Male Sexual Dysfunction: Erectile dysfunction and premature ejaculation. European Association of Urology 2015 [cited 2018 May 28]; Available from: https://uroweb.org/wp-content/uploads/EAU-Guidelines-Male-Sexual-Dysfunction-2015-v2.pdf. | 16 (45.7%) |
| 18 | Stupp R, Brada M, van den Bent MJ, Tonn JC, Pentheroudakis G. High-grade glioma: ESMO Clinical Practice Guidelines for diagnosis, treatment and follow-up. Annals of oncology. 2014;25 Suppl 3:iii93-101. | 15 (42.9%) |
| 19 | Fizazi K, Greco FA, Pavlidis N, Daugaard G, Oien K, Pentheroudakis G. Cancers of unknown primary site: ESMO Clinical Practice Guidelines for diagnosis, treatment and follow-up. Annals of oncology. 2015;26 Suppl 5:v133-8. | 12 (34.3%) |
| 20 | Cardoso F, Costa A, Norton L, Senkus E, Aapro M, Andre F, et al. ESO-ESMO 2nd international consensus guidelines for advanced breast cancer (ABC2). Breast. 2014;23(5):489-502. | 19 (54.3%) |
| 21 | Biegstraaten M, Arngrímsson R, Barbey F, Boks L, Cecchi F, Deegan PB, et al. Recommendations for initiation and cessation of enzyme replacement therapy in patients with Fabry disease: the European Fabry Working Group consensus document. Orphanet Journal of Rare Diseases. 2015;10:36. | 20 (57.1%) |
| 22 | Weimann A, Braga M, Harsanyi L, Laviano A, Ljungqvist O, Soeters P, et al. ESPEN Guidelines on Enteral Nutrition: Surgery including organ transplantation. Clinical nutrition. 2006;25(2):224-44. | 18 (51.4%) |
| 23 | Malfertheiner P, Megraud F, O'Morain CA, Atherton J, Axon AT, Bazzoli F, et al. Management of Helicobacter pylori infection--the Maastricht IV/ Florence Consensus Report. Gut. 2012;61(5):646-64. | 19 (54.3%) |
| 24 | Umpierrez GE, Hellman R, Korytkowski MT, Kosiborod M, Maynard GA, Montori VM, et al. Management of hyperglycemia in hospitalized patients in non-critical care setting: an endocrine society clinical practice guideline. The Journal of clinical endocrinology and metabolism. 2012;97(1):16-38. | 19 (54.3%) |
